# Synergy between ketamine and ketogenic diet in anorexia nervosa, and other neurobehavioral disorders

**DOI:** 10.1007/s40519-023-01528-5

**Published:** 2023-02-14

**Authors:** Barbara Scolnick, Caroline Beckwith

**Affiliations:** 1 Internal Medicine, 11 Irvington Street, Waban, MA 02468 USA; 2 Recovery Coach, 2621 Palisades Avenue, Bronx, NY 10463 USA

## Abstract

Through serendipity, we observed an apparent synergy between adopting a ketogenic diet and receiving ketamine infusions that led to complete and durable remission in two patients with chronic enduring anorexia nervosa. Both patients had struggled with the disorder for over a decade. We offer a hypothesis, based on glutamate to explain this synergy, and hope it stimulates further research.

This hypothesis arises from clinical observation. In 2021, a young woman with a 15-year history of chronic anorexia nervosa experienced a complete and lasting remission after adopting a ketogenic diet, followed by intravenous ketamine [1]. This unorthodox treatment was enabled, because the patient was the niece of the treating physician. In light of the startlingly positive response, we conducted a pilot study of five patients with chronic anorexia nervosa. One patient who had been ill for decades followed a similar clinical course and remains in complete remission [2]. Of note, patient one had been diagnosed with chronic anorexia nervosa, intermittent alcohol use disorder and mild depression. She had been sober for 12 months prior to the unique treatment, and had not been on any medication. Patient two had received diagnoses of chronic anorexia and intermittent moderate depression. At the time of treatment, she had been on bupropion 300 mg/day and sertraline 100 mg twice/day for over 2 years. These two “super-responders” led us to wonder if there is biological mechanism for synergy between the ketogenic diet and ketamine infusion. Our hypothesis centers on the glutamate system.

Briefly, glutamate is the major excitatory neurotransmitter in the brain. Its metabolism is complex and marked by a glutamine/glutamate cycle. The essence of this cycle is that neurons do not synthesize glutamate; rather astrocytes absorb glutamate from the synapse; convert this into glutamine and release glutamine to neurons, which then go on to convert glutamine to glutamate, release it to the synapse, and the cycle turns again [3]. It is important to recognize that glutamate exists in dynamic equilibrium with alpha ketoglutarate and oxaloacetate. Alpha ketoglutarate and oxaloacetates are key ketoacids in the Krebs cycle, the highly evolutionarily conserved mechanism involved in ATP synthesis in the cell.

The ketogenic diet has been an effective treatment for seizure disorder for over 100 years, yet the reasons for its anti-seizure effects are unclear. Because gamma aminobutyric acid (GABA) a neuroinhibitory transmitter is linked to the glutamate/glutamine cycle, it has been suggested that during ketosis, the switch in brain energy substrate from glucose to ketone bodies leads to these substrates entering the Krebs cycle at the acetyl Co A step, rather than the “usual” glucose to pyruvate glycolysis. This increases flux through the Krebs cycle, which, in turn, increases demand for ketoacids in the Krebs cycle and increases production of glutamate, and ultimately increases GABA which acts as an anti-seizure agent [3].

Since 1983, the “dissociative anesthetic” ketamine has been proven to be a glutamate N-methyl-D-aspartate (NMDA) receptor antagonist, thus invoking the glutamate system as involved in its myriad effects. Ketamine is increasingly being used to treat major depression, with encouraging results.

The pathogenesis of anorexia nervosa remains unknown, but more sophisticated and discriminating techniques of magnetic resonance spectroscopy allow direct interrogation of the glutamate system in the brain. A recent case/control study documented decreased glutamate in patients with AN compared with controls in the anterior cingulate, occipital cortex, and putamen regions [4]. It is difficult to unequivocally determine whether the dramatic response seen in these two patients was due to a beneficial effect on their underlying depressive syndrome which then caused improvement in AN symptoms; or on the contrary, the AN disorder directly improved which then caused improvement on the underlying depression symptoms. We strongly think that it was the latter. Both patients said that they were relieved that the “anorexic voice” which had tormented them for decades was silenced. Both patients stated that the freedom from anorexia was the main effect of the treatment. Both patients, although having struggled with anorexia nervosa for decades, had only mild depression in patient one, and moderate but intermittent depression in patient two.

Chronic anorexia nervosa is a tragic disorder with no known treatment. These results, although limited, are striking. We do not know if there is synergy between ketamine and ketogenic diet with respect to major depression. However, the fact that many patients fail to respond to ketamine alone, or respond for only a short time, should motivate studies of possible synergy.

We hope that this letter stimulates such research.
